# A single polyploidization event at the origin of the tetraploid genome of *Coffea arabica* is responsible for the extremely low genetic variation in wild and cultivated germplasm

**DOI:** 10.1038/s41598-020-61216-7

**Published:** 2020-03-13

**Authors:** Simone Scalabrin, Lucile Toniutti, Gabriele Di Gaspero, Davide Scaglione, Gabriele Magris, Michele Vidotto, Sara Pinosio, Federica Cattonaro, Federica Magni, Irena Jurman, Mario Cerutti, Furio Suggi Liverani, Luciano Navarini, Lorenzo Del Terra, Gloria Pellegrino, Manuela Rosanna Ruosi, Nicola Vitulo, Giorgio Valle, Alberto Pallavicini, Giorgio Graziosi, Patricia E. Klein, Nolan Bentley, Seth Murray, William Solano, Amin Al Hakimi, Timothy Schilling, Christophe Montagnon, Michele Morgante, Benoit Bertrand

**Affiliations:** 1 IGA Technology Services S.r.l., via Jacopo Linussio 51, I-33100 Udine, Italy; 2 World Coffee Research, 5 avenue du grand chêne, 34270 Saint-Mathieu-de-Tréviers, France; 3 Istituto di Genomica Applicata, via Jacopo Linussio 51, I-33100 Udine, Italy; 4 University of Udine, Department of Agricultural Food, Environmental and Animal Sciences, via delle scienze 206, I-33100 Udine, Italy; 5 Institute of Biosciences and Bioresources, National Research Council, via Madonna del Piano 10, I-50019, Sesto Fiorentino (FI), Italy; 6 Luigi Lavazza S.p.A., Innovation Center, I-10156 Torino, Italy; 7 Illycaffè S.p.A., Research & Innovation, via Flavia 110, I-34147 Trieste, Italy; 8 Department of Biotechnology, University of Verona, Verona, Italy; 9 CRIBI, Università degli Studi di Padova, viale G. Colombo 3, I-35121 Padova, Italy; 10 Department of Life Sciences, University of Trieste, I-34148 Trieste, Italy; 11 Department of Horticultural Sciences, Texas A&M University, College Station, TX USA; 12 Department of Soil and Crop Sciences, Texas A&M University, College Station, TX USA; 13 CATIE, Turrialba, Costa Rica; 14 Faculty of Agriculture, Sana’a University, Sana’a, Yemen; 15 CIRAD, IPME, 34 398 Montpellier, France; 16 UMR IPME, Univ. Montpellier, IRD, CIRAD, 34 398 Montpellier, France

**Keywords:** Computational biology and bioinformatics, Evolution, Genetics, Plant sciences

## Abstract

The genome of the allotetraploid species *Coffea arabica* L. was sequenced to assemble independently the two component subgenomes (putatively deriving from *C. canephora* and *C. eugenioides*) and to perform a genome-wide analysis of the genetic diversity in cultivated coffee germplasm and in wild populations growing in the center of origin of the species. We assembled a total length of 1.536 Gbp, 444 Mb and 527 Mb of which were assigned to the canephora and eugenioides subgenomes, respectively, and predicted 46,562 gene models, 21,254 and 22,888 of which were assigned to the canephora and to the eugeniodes subgenome, respectively. Through a genome-wide SNP genotyping of 736 *C. arabica* accessions, we analyzed the genetic diversity in the species and its relationship with geographic distribution and historical records. We observed a weak population structure due to low-frequency derived alleles and highly negative values of Taijma’s *D*, suggesting a recent and severe bottleneck, most likely resulting from a single event of polyploidization, not only for the cultivated germplasm but also for the entire species. This conclusion is strongly supported by forward simulations of mutation accumulation. However, PCA revealed a cline of genetic diversity reflecting a west-to-east geographical distribution from the center of origin in East Africa to the Arabian Peninsula. The extremely low levels of variation observed in the species, as a consequence of the polyploidization event, make the exploitation of diversity within the species for breeding purposes less interesting than in most crop species and stress the need for introgression of new variability from the diploid progenitors.

## Introduction

*Coffea arabica* is an allopolyploid species (*2n = 4x* = 44) resulting from the hybridization between two species most closely related to *C. eugenioides* and *C. canephora*^1^. The allopolyploid speciation of *C. arabica* has a broad time interval estimate spanning 10,000 or 665,000 years BP^2,3^. Unlike many tropical tree crops, Arabica coffee is not clonally propagated. Cultivars and landraces are typically propagated by seed. The mating system is primarily based on self-fertilization, although pollinator-mediated outcrossing may occasionally occur. The predominant autogamy leads to high levels of inbreeding.

*C. arabica* is indigenous to Ethiopia and South Sudan which represents its primary center of diversity^4^ (FAO-1964, ORSTOM-1966). Yemen is a secondary dispersal center^5^. Several accounts of the early history of *C. arabica* germplasm usage and movements are available in the literature, but the most complete and best documented publication is the one of Haarer (1958). Sometimes during the 14^th^ century, coffee seeds were brought out of the forests of South Western Ethiopia to Yemen, where coffee cultivation expanded to satisfy the demand of a growing number of coffee houses in Moccha and Cairo at the end of the 15th century. The center of diversity in South Western Ethiopia corresponded to the area that has been for centuries under the control of the Kingdom of Kafa. This area was described as an impenetrable “citadelle” until Menelik II conquered the Kingdom in 1897. Hence, early escapes of coffee seeds from South Western Ethiopia were likely occasional and the coffee cultivation in Yemen has started from a narrow genetic basis. *C. arabica* was then spread worldwide from Yemen rather than from its primary center of origin in Ethiopia. Significant out-of-Yemen movements were documented (i) in 1670, from Yemen to India with some seeds smuggled by Baba Budan and (ii) in 1715, from Yemen to Bourbon Island (today Ile de la Réunion) with very few seeds. The former gave rise to the Typica variety after the Dutch brought some seeds from India to today’s Indonesia in 1696 and 1699. From Indonesia through Europe, Typica reached the Americas in 1723. The latter gave rise to the Bourbon variety that reached the Americas and East Africa in the mid-19th century. In India, the early seeds introduced in 1670 were grown locally for centuries. In the early 20th century, East Africa (today Burundi, Rwanda, DR Congo, Kenya, Tanzania and Uganda) started coffee cultivation and introduced seeds from Yemen, Bourbon, Typica and Indian varieties and very few Ethiopian landraces, which leaked from Ethiopia in the pockets of travelers (Geisha and Rume Sudan for instance). Hence, East Africa could be considered as a melting pot of all different varieties descending from early coffee cultivation in Yemen^6^.

It is evident that molecular analyses of genetic diversity are needed to support this scenario that is based on geographical and historical data. In the past, microsatellites or RAPDs have been used for studying genetic diversity in *C. arabica*^7–9^. More recently, a molecular study of *C. arabica* germplasm was performed by Merot-L’anthoene *et al*.^10^ using an 8.5k SNP array, which represents the first genome-wide analysis of the genetic diversity in this species but it suffers from the ascertainment bias associated with the limited breadth of variation included in the SNP discovery panel. Unbiased approaches to investigate genetic diversity are supposed to rely on the availability of a *C. arabica* genome sequence. The genome of a modern accession of *C. canephora*, one of the progenitors of *C. arabica*, has been sequenced^11^, but the assembly of a diploid genome provides limited support for analyzing sequencing data from tetraploid coffee germplasm. Genome sequencing initiatives of tetraploid accessions have been launched by several research groups (https://coffeegenome.ucdavis.edu/^12^, among others) but an open-access genome assembly, with a reliable sorting of homoeologous sequences, is not yet available. Decoding the allotetraploid genome of *C. arabica* is therefore mandatory to have accurate genotyping-by-sequencing (GBS) studies in this species.

One of the challenges in short read sequencing of polyploid genomes is the difficulty in assembling a reference sequence for the haploid complement by disentangling its homoeologous components. Here we used short read sequencing of pooled BAC clones. Each DNA pool contained ~3% of the haploid genome and was sequenced and assembled separately. This strategy resulted into the first public draft genome of *C. arabica* L. that enabled us to undertake the first GBS approach for polymorphism detection on *C. arabica* accessions and its parental species. This allowed us to (i) confirm the geographical structure of the genetic diversity of *C. arabica* which originated after a single event of polyplodization that gave rise to the species and was partly shaped by early movements of planting material, (ii) detect sources of diversity for coffee breeding and (iii) understand the relatedness between the canephora subgenome of *C. arabica* and the modern diploid *C. canephora*, some of which are used for the production of Robusta coffee beans.

For this analysis we selected *C. arabica* varieties conserved in the CATIE International Coffee Collection in Costa Rica. This collection does not only contain elite cultivars, but also the most extensive sampling of *C. arabica* genetic diversity available outside of Ethiopia. We studied also 93 Yemeni genotypes collected from farmers’ fields in Yemen. Based on the GBS analysis of 736 *C. arabica* accessions we developed a description of the genetic diversity of the species in the context of their geographical distribution and historical records.

## Results

### Genome sequencing and assembly

A BAC library was constructed from a *C. arabica* plant of the variety ‘Bourbon Vermelho’. BAC clones amounting to a ~2.8X genome coverage were arranged into 96 pools of 384 clones (Table S1). DNA pools were sequenced independently, generating 488 Gbp that were assembled using ABySS v1.3.7^13^. We also produced for the same individual 42.7 Gbp from genome-wide mate pairs spanning 2 kbp DNA fragments (Table S1). Such mate pairs were used for scaffolding BAC contigs, resulting in 164,254 scaffolds, with an N50 of 19,010 and an L50 of 22.3 kbp, amounting to a total length of 1.536 Gbp. Based on k-mer analysis of paired-end whole genome shotgun (WGS) sequences, we estimated a ~1.3 Gbp genome size. For low values of k, *e.g*. k = 16, we observed a bimodal distribution of coverage in *C. arabica* (Fig. S1). One peak pointed to a value of ~54X, corresponding to the expected mean genome coverage after read trimming and filtering. Another peak pointed to a 2-fold higher coverage that is expected when sequences are identical between subgenomes. We did not observe any peak corresponding to half of the diploid genome coverage that would have been expected in the presence of substantial levels of heterozygosity, compatible with the self-fertilizing nature of *C. arabica*. Conversely, for higher values of k, *e.g*. k = 51, the k-mer analysis produced a single peak exactly at the expected genome coverage, indicating a relatively high level of diversity between the two subgenomes. We then generated 38 Gbp of short reads from a *C. eugenioides* accession, corresponding to a coverage of approximately 54X (Table S1), while raw reads from a *C. canephora* doubled-haploid accession DH200-94^11^ corresponding to a 66X coverage were downloaded from the NCBI Sequence Read Archive (SRA). After masking repetitive sequences from *C. arabica* scaffolds we compared 51-mers generated from each scaffold with 51-mers obtained from *C. eugenioides* and *C. canephora* WGS reads. A total of 25,315 scaffolds, amounting to a total length of 444 Mbp, shared more 51-mers with *C. canephora* than with *C. eugenioides* and were therefore assigned to the canephora subgenome. A total of 26,627 scaffolds, amounting to a total length of 527 Mbp, shared more 51-mers with *C. eugenioides* than with *C. canephora* and were therefore assigned to the eugenioides subgenome. The remaining 112,312 scaffolds, amounting to a total length of 565 Mbp, could not be assigned with high confidence to either subgenome due to either their short size or the presence of repetitive sequences or the high similarity between homoeologous sequences. We sequenced 560 million reads, corresponding to 70 Gb of RNA-seq from 8 different tissues of *C. arabica*, ‘Bourbon Vermelho’ variety (Table S1 and section methods). We used these sequences to predicted 46,562 non-redundant gene models in the *C. arabica* genome that include 92.4% of the plant orthologs set of BUSCO^14^, see Supplementary Material for details. Of these, 21,254 and 22,888 genes were located on scaffolds assigned to the canephora and eugenioides subgenomes, respectively, while 2,420 genes were located on unassigned scaffolds. Gene predictions are downloadable from the website https://worldcoffeeresearch.org/work/coffea-arabica-genome/.

**Figure 1:**
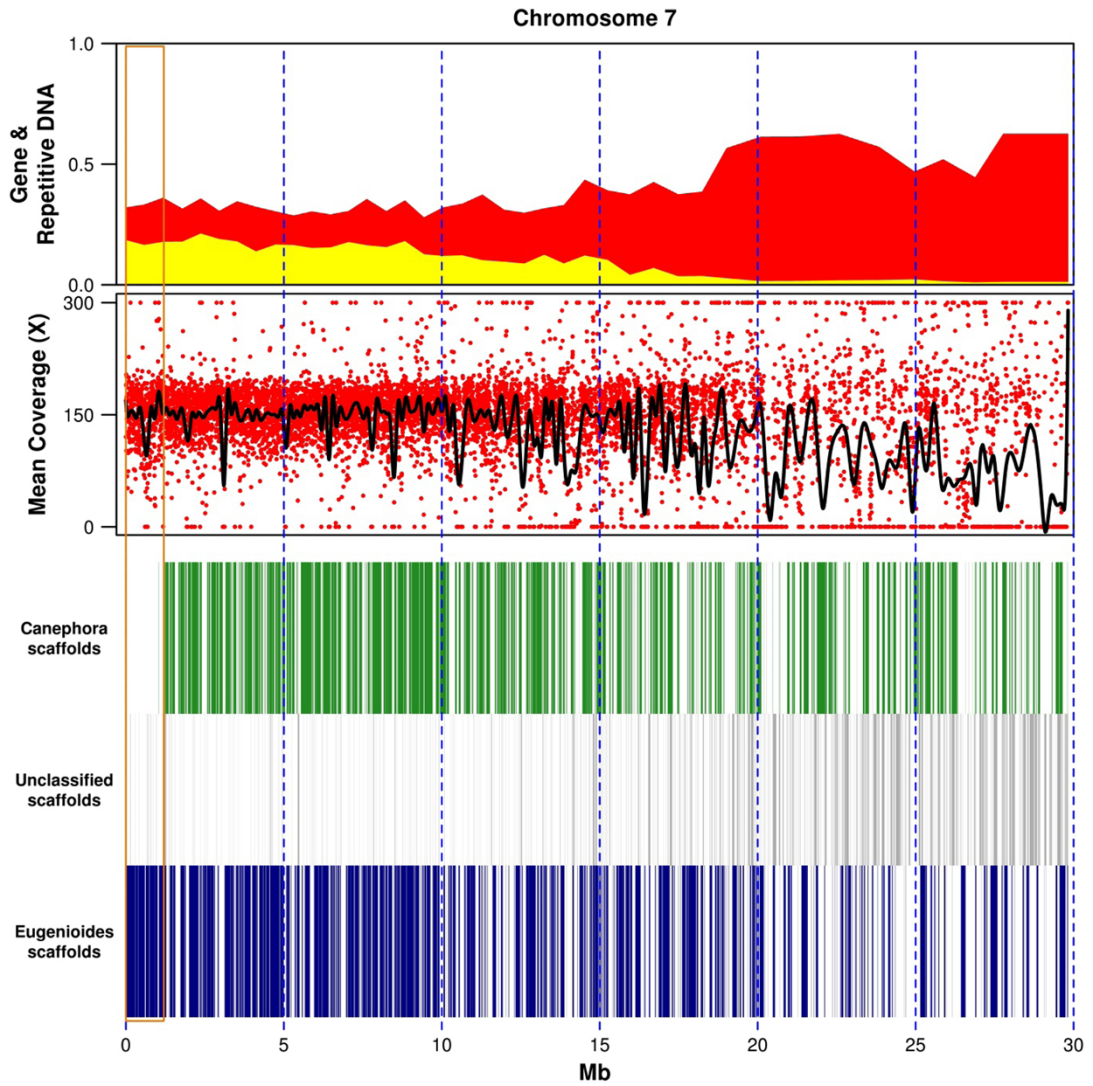
Homoeologous replacement on chromosome 7. The brown rectangle indicates a large event of homoeologous replacement. The upper plot indicates the fractions of nucleotides in 100-kb non-overlapping windows classified as gene space (yellow), repetitive DNA (red), and intergenic low-copy DNA (white background). The lower plots illustrate the average read coverage in 2-kb non-overlapping windows (red dots) and a cubic smoothing spline of the data (black line), the alignment of *C. arabica* scaffolds, sorted by their subgenome assignment (canephora in green, eugenioides in blue) against the genome reference of the diploid *C. canephora*.

The tetraploid genome in the sequenced accession of ‘Bourbon’ did not show major chromosomal deletions compared to a reference diploid genome of one of the progenitor species (*C. canephora*). We detected a single large chromosomal rearrangement, corresponding to an event of homoeologous replacement of canephora DNA with eugeniodes DNA in the terminal 1.2-Mbp of chromosome 7, involving 179 predicted genes (Fig. 1), in agreement with previous findings based on the lack of inter-homeologue polymorphisms (hemi-SNP) in that region^15^. ‘Bourbon’ is therefore an autopolyploid across this gene-rich region, carrying four copies of eugenioides DNA that likely originated through a homology-directed repair of a double-strand break in the canephora chromosome. This event likely occurred immediately after hybridization because we did not detect hemi-SNP across that region in any accession of *C. arabica* analyzed in this study. We selected approximatively 1 Mbp of shared nucleotide sequence between the canephora and eugeniodes subgenomes in ‘Bourbon Vermelho’, using the largest scaffolds that contained single-copy genes (BUSCO). Shared regions within each scaffold were identified using (B)LastZ and realigned using MUSCLE. Nucleotide diversity (π) between *C. arabica* subgenomes based on hemi-SNPs amounted to 3.1 × 10^−2^.

### *Coffea arabica* genetic diversity

We used GBS data to estimate genetic diversity in a sample of 736 accessions of *C. arabica* (Dataset S2), presumably representing a large fraction of the diversity available in the species. The nucleotide diversity estimate obtained is low (π = 2.3 × 10^−4^, based on 652 SNPs across 193,873 informative nucleotides), a value that is one order of magnitude lower than that estimated in the present-day germplasm of the two progenitor diploid species (*C. canephora* π = 2.6 × 10^−3^*, C. eugenioides* π = 1.1 × 10^−3^). We observed a very peculiar distribution of private mutations (*i.e*. variant sites detected in a single individual only), with such mutations corresponding to 74.4% of the variant sites. In order to exclude an underestimation of π due to any alignment bias between the native and derived subgenomes used as a reference genome in our procedure of read mapping (see Materials and Methods for more details) or due to the fragmentation of our reference, we repeated this analysis by aligning dRAD reads against a chromosome-scale assembly of *C. arabica* ‘Caturra Vermelho’ that became recently available (GenBank assembly accession: GCA_003713225.1). We confirmed the levels of π both in the progenitor diploid species (*C. canephora* π = 2.3 × 10^−3^*, C. eugenioides* π = 1.1 × 10^−3^) and *C. arabica* (π = 2.0 × 10^−4^) across a larger sample of 339,526 nucleotides.

The value of Taijma’s *D* in *C. arabica* was highly negative (−2.51), as expected for a population undergoing expansion after a recent severe bottleneck. A large fraction (87.6%) of variant sites in *C. arabica* showed a minor allele frequency lower than 0.05. Among the variant sites that were present in more than one individual of *C. arabica*, 28.7% did not violate Hardy-Weinberg equilibrium, 6% showed an excess of heterozygotes and the majority (65.3%) showed a deficiency of heterozygotes, as expected for an autogamous species.

**Figure 2:**
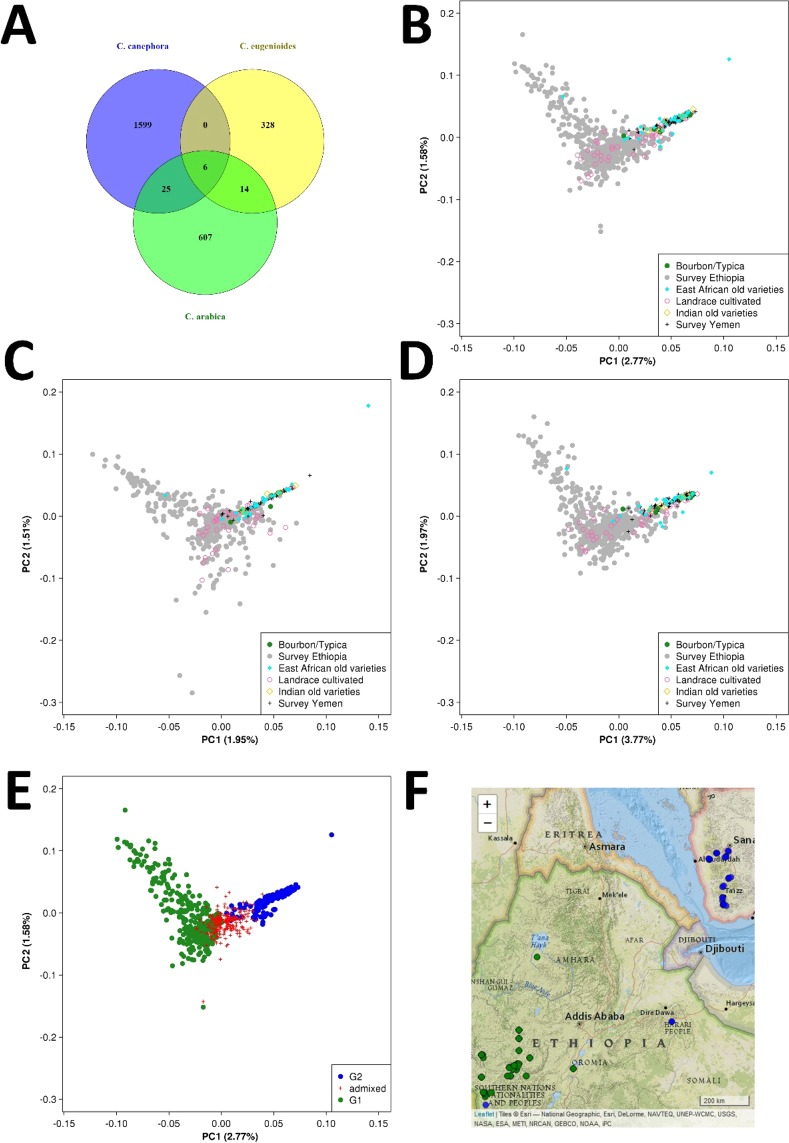
Genetic diversity in *Coffea*. Number of variant sites and their distribution among the three species (panel A), number of individuals n = 53 in *C. canephora*, n = 10 in *C. eugenioides*, n = 736 in *C. arabica*. Principal Component Analysis within *C. arabica* using all variant sites (panel B) or only variant sites on the canephora (panel C) or on the eugeniodes subgenome (panel D), separately. Individuals were grouped *a priori* into geographical classes. Principal Component Analysis of the Arabica populations (panel E) using all variant sites, as in panel B, in relation to ancestry assignment provided by the software STRUCTURE. Map of Ethiopia and Yemen showing the locations of coffee accessions (panel F), as a function of their STRUCTURE population, as in panel E.

The severity of the bottleneck effect and the unsubstantial carry-over of ancestral diversity from the *Coffea* genus, represented in this study by the most likely parental species, into the population of *C. arabica* suggest that the tetraploid species has originated from a single event of hybridization. This conclusion is supported also by the distribution of private alleles among the three species (*C. arabica*, *C. canephora*, *C. eugenioides*) that shows that the vast majority of SNPs identified in *C. arabica* (Fig. 2A) is not shared with either of the parental species, confirming that most of the variation present today in *C. arabica* has arisen after the polyploidization event and also indicating that there have not been major introgression events from the two parental species into *C. arabica*.

We performed *in silico* simulations with msprime^16^ to assess whether diversity levels and distribution are compatible with a recent origin of all current *C. arabica* accessions from a single polyploid individual. We simulated mutation accumulation with mutation rates ranging from 6.5 × 10^−10^ to 3.25 × 10^−8^ per base per generation, and under four alternative hypothetical demographic models (Dataset S1) of a current population of effective size *N*_*e*_ = 10,000 or 50,000 initiated with a single hybrid individual 10,000 or 20,000 years ago, which attained the final size in 200 generations of exponential growth followed by a constant size or recovering to the final size from a bottleneck that occurred 1,000 years ago. The other parameters in the model were kept constant (recombination rate 1 × 10^−8^, generation per year = 0.2, sampling size = 700 individuals). These models predicted a nucleotide diversity π ranging between 1.2 × 10^−4^ and 2.5 × 10^−4^ for the highest mutation rate and between 2.4 × 10^−6^ and 5 × 10^−6^ for the lowest mutation rate and a proportion of private SNPs ranging from 23% (polyploidization 10,000 years ago, Ne =10,000) to 35% (polyploidization event 10,000 years ago, Ne =50,000, Dataset S1). Considering a generation time of 5 years, the highest mutation rate per generation of 3.25 × 10^−8^, that provides diversity estimates that are very similar to those observed, corresponds, on a per year basis, to that estimated in Arabidopsis^17^. Since coffee varieties and landraces are propagated by seed for establishing new plantations, we also simulated a recent expansion since the beginning of massive coffee cultivation (approx. 0.4 kya) to a current size of N_e_ = 500,000. This model did not predict substantial changes in the expected values of π but it generated higher expected values of private SNPs (42%) that are closer to the observed values.

Under the assumption of a recent origin of all *C. arabica* accessions from a single individual that underwent polyploidization, that seems to be fully supported by both the available data and the *in silico* simulations, expectations can be derived on the patterns and distribution of genetic diversity within the *C. arabica* population itself and in comparison to diversity within the ancestral species.

**Figure 3:**
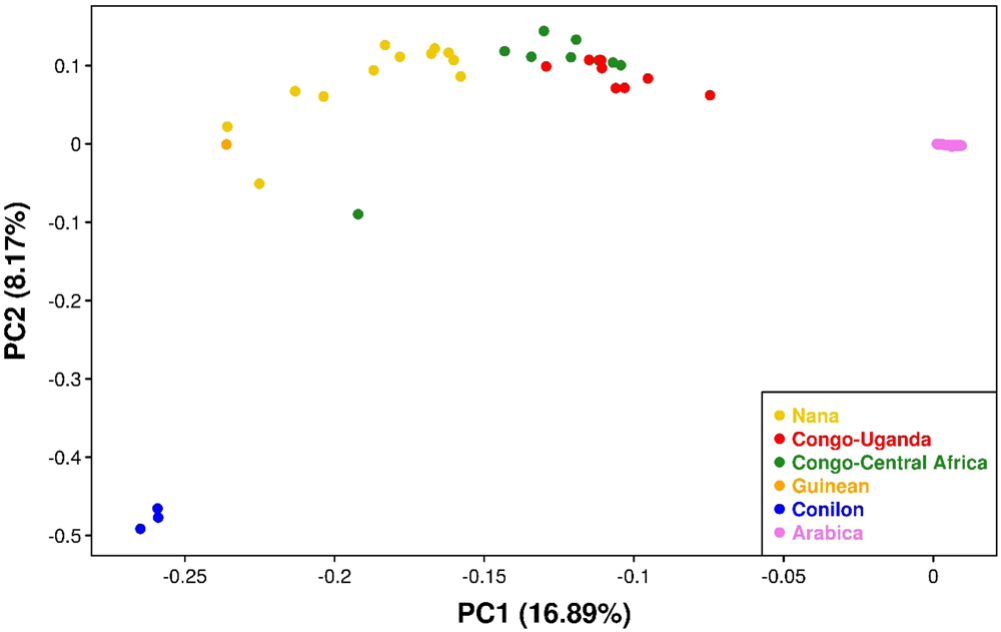
Principal Component Analysis for Arabica and canephora individuals using only the variants found in the C. *canephora* subgenome. Analysis of the first two axes with the percent variation explained (Nana group, Robusta Congo-Uganda group, Robusta Congo Central Africa group, Guinean group and Conilon group are represented in yellow, red, green, orange and blue, respectively).

We analyzed diversity within *C. arabica* in relation to diversity in 35 *C. canephora* accessions using only the variants identified in the canephora subgenome by performing a Principal Component Analysis (PCA) where the first two PC’s explained 16.9 and 8.2% of the variance (Fig. 3).

The *C. canephora* accessions are representative of the diversity within the species including what is commercially identified as “Conilon”, *e.g. C. canephora* accessions used for coffee production in Brazil, and “Robusta”, *e.g. C. canephora* accessions cultivated elsewhere in the world^18^. The genetic diversity of the modern *C. canephora* is much wider than the one of the canephora subgenome of *C. arabica*, as expected under the single polyploidy event scenario. The Robusta groups of modern *C. canephora* from Congo-Central Africa and Congo-Uganda appear to be the closest to the lineage that has donated the canephora subgenome to *C. arabica*, confirming the observations based on a SNP chip array analysis^10^. Interestingly, in this PCA, the “Conilon” group is clearly separated from other *C. canephora* groups that instead form a continuum.

A principal component analysis was then conducted solely in *C. arabica* individuals using 698 variant sites identified across 129,638 informative positions over the two subgenomes (Fig. 2B) or using separately variant sites on either subgenome (Fig. 2C,D). The structure observed was very similar whether or not subgenome-specific SNPs were merged; nevertheless, the percentages explained by the first two components, that are always very low, were slightly higher when using the eugenioides subgenome, meaning that using the genetic variation across this subgenome enables a better description of the structure of the population. As expected under a scenario where the polymorphisms are extremely recent and mostly private, the substructuring of the population appears very weak, even though still detectable. The first axis (PC1) separates the majority of the Ethiopian population (Landrace cultivated, Survey Ethiopia) from the Yemeni population (other geographical classes, Dataset S2) but some Ethiopian genotypes were located close to the Yemeni category and *vice versa* (Fig. 2), as expected based on a recent origin (dating to the 14^th^ century) of the Yemeni population from Ethiopian accessions (Chevalier 1929). Indian old varieties and East African old varieties, as well as Bourbon/Typica varieties, overlapped with the area of the PCA plane populated by Yemeni germplasm (Fig. 2). The group ‘Landrace cultivated’ covered entirely the area of the PCA plane populated by Yemeni germplasm and only partially the area of the PCA plane populated by Ethiopian germplasm, suggesting that part of the genetic diversity present in Ethiopia is currently used for cultivation only locally and another part has not been exploited yet. The separation between widely cultivated varieties and part of the Ethiopian wild germplasm was corroborated by ancestry assignment performed with the software STRUCTURE. At K = 2, one ancestry group (G1) included the vast majority of *C. arabica* accessions that were either surveyed in Ethiopia or were recently brought out of Ethiopia (Fig. 2E,F, Table S2,: Fig. S2) and the other group (G2) included all the Yemeni varieties, their descendants and a few Ethiopian accessions grown in the easternmost part of the country. By running STRUCTURE only on G1 a clear structuration of the Ethiopian germplasm was revealed (Additional text).

## Discussion

Whole genome sequencing is revolutionizing our understanding of biology and the field is developing rapidly due to advances in DNA sequencing technologies. The accuracy of genome sequencing and assembly of complex genomes is affected by different factors ranging from plant material availability to genome size, GC content, repeat content, ploidy level, sequencing technology, and bioinformatic software^19–22^. Long-read sequencing technologies, such as those of Pacific Biosciences or Oxford Nanopore Technologies, are currently used for reconstructing Mb-long chromosome sequences or both haplotypes of highly heterozygous genomes^23–27^. Short-read sequencing is less expensive, provides more coverage with the same investment and thus more sequence accuracy, but it leads to more fragmented assemblies. Several applications such as the analysis of gene content, the discovery and application of genetic markers and studies of genetic diversity can be undertaken using draft whole genome shotgun assemblies, which are relatively quick to produce. They allow to reconstruct efficiently the non-repetitive fraction of the genome, which is the most informative for those applications. In our study we adopted a strategy to maximize the accuracy of sequence assembly, with the aim of reconstructing separately homoeologous sequences and provide a reference for studying allelic variation in *C. arabica*. Our genome assembly is relatively fragmented with an L50 of 22.3 kbp but it contains 92.4% of the conserved set of plant single-copy orthologs (BUSCO) and allowed us to assign 94.8% of the predicted genes to their parental genome. We predicted 46,562 protein‐coding genes in *C. arabica*, which is nearly double the number (25,574) of one of its ancestor *C. canephora*^11^. This high number is fully compatible with the hypothesis of a recent genome doubling and limited gene loss or pseudogenization since the event of polyploidisation to the present time.

Given the extremely low level of heterozygosity of *C. arabica*, the main issue we had to face was the ploidy level^28^. To tackle this, we could have opted to isolate chromosomes through flow cytometry^29^ but we could not consider this technology due to lack of appropriate genetic stocks that would allow for easy chromosome isolation. We thus opted for a hierarchical sequencing approach of BAC pooling where the likelihood of two homoeologous fragments occurring in the same pool was extremely low^30,31^, leading to dramatic reduction of the genome complexity in the assemblies of each pool. In this paper, we show a successful application of this genome assembly for studying Arabica diversity.

Coffee is a recent beverage relative to the time scale of mankind. Ethiopian nomadic mountain people were probably the first to recognize coffee’s stimulating effect. However, it is estimated that the use of coffee brewing as we know it today would have begun in the Middle Ages in Yemen. The first archaeological evidence of beverage coffee consumption was found in Zabid (Yemen) a city at the south end of the Arabian Peninsula^32^. Yemen would have become the first coffee market. In Ethiopia, drinking coffee was formally prohibited for Christians until the early 20th century and the Ethiopian Coffee ceremony is considered a recent invention^33^. The prevailing assumption is that coffee seeds were introduced from Ethiopia to Yemen. From southwestern Ethiopia, wild coffee genotypes should have been acclimated to the other side of the gulf. However, domestication of *C. arabica* over a long period of time (end of 17th century) has also been reported in Ethiopia by scientific observers or travelers in the Harar region, Zeghie peninsula, Sidamo, and Welega province^34^. During the 15th and 16th centuries, coffee cultivation was developed within Yemen to meet local needs. In Yemen, people transformed the mountainsides into terraced hillsides, built irrigation networks, and invented farming techniques including growing coffee without shade. In our study we compared some ‘Typica’ and ‘Bourbon’–derived cultivars with the Yemeni and Ethiopian accessions. In Latin America, breeders exploited these two narrow genetic bases, which resulted in ‘Typica’ and ‘Bourbon’–derived cultivars, all showing similar agronomic traits and high susceptibility to the major coffee diseases and pests^35^. Today, over 80% of Arabica coffee is produced in Latin America and Arabica coffee production is still based to a large extent on cultivars developed long ago by line selection within the ‘Typica’ and ‘Bourbon’ varieties or among seedlings originating from crosses between these varieties^36^.

We have chosen to analyze genetic diversity and population divergence within the *C. arabica* species and in comparison to the ancestral donor species using an unbiased resequencing-based method such as GBS rather than using a SNP chip such as the one recently developed for coffee^10^. Even though we have analyzed a total number of SNPs that is lower than that present on the above mentioned chip, the unbiased view of sequence diversity provided by the GBS approach has allowed us, on one hand, to obtain robust estimates of nucleotide diversity, allele frequency spectra and other population parameters that could be used to infer past population demographic history and, on the other hand, to avoid the ascertainment bias in estimating population divergence that is intrinsic to SNP chip-based approaches caused by the limited number of individuals from specific populations present within the initial SNP discovery panels^37,38^.

We found in *C. arabica* the lowest level of genetic diversity reported so far in crop species (Table S4), only comparable to levels observed for bread wheat, another recent allopolyploid species, and an exceedingly large fraction of private alleles, both to individual accessions as well as to the species in comparison to the progenitor species. All evidence collected, including that from forward computer simulations of mutation accumulation, are compatible with the origin of *C. arabica* from a single polyploidization event that occurred in very recent evolutionary times. This is the simplest model for allopolyploid evolution (Doyle and Egan 2010) and predicts that all variation observed in the new species is due to new mutations arisen after the polyploidization event. During the early stages following polyploid formation, the occurrence of a ‘genomic shock’ leading to gene loss and/or homeologous recombination has been observed in some species^39^. In *C. arabica* we observed a single instance of homeologous replacement at the tip of chromosome 7 and thus we do not have evidence of a significant contribution of major structural rearrangements occurring after polyploidization.

Despite the hypothesized very recent origin of all accessions from a single allopolyploid and the consequent very recent origin of all polymorphisms surveyed, our results revealed genetic differentiation between Ethiopian accessions, still present in rainforest areas at the Southwesternmost range of distribution of the species, and the germplasm used for intensive plantation in Eastern Ethiopia and in Yemen that is also genetically similar to all cultivated varieties worldwide, from East Africa and India as well as belonging to the Bourbon/Typica lineages. These results are in agreement with historical information on a worldwide spreading of *C. arabica* out of Yemen through the Bourbon/Typica lineages and point to the existence of wild Ethiopian germplasm that could represent a yet untapped, even though very limited, reservoir of novel genetic variation for improvement of *C. arabica* cultivated varieties (see supplementary materials for a more detailed analysis of variation patterns within the Ethiopian population).

In the study of Lashermes *et al*.^40^, the authors used RAPD markers to genotype 20 accessions, observed two groups, consisting of either cultivated coffee or wild Ethiopian accessions. A similar study^7^ with 119 accessions and 16 markers showed similar results. Finally, a study by Silvestrini *et al*.^41^ based on 73 accessions and 15 SSR markers found only two groups, the cultivated group from Yemen and a second group representing accessions from Ethiopia.

Genetic diversity is the foundation of the genetic improvement of crops and has become a strategic economic and cultural identity issue. The challenges of *C. arabica* germplasm conservation in Ethiopia have been reviewed by Labouisse *et al*.^42^ and regional human overpopulation appears to be the main cause of accelerated destruction of the biodiversity in the montane forests of southwestern Ethiopia^43^. Knowledge of coffee genetic diversity at the molecular level is essential for effective strategic conservation in *ex situ* collections and for protection of *in situ* populations, as well as the use of these resources to meet both current and future breeding needs. The vast germplasm collection of CATIE is easily accessible but its use for crop improvement is still very limited. Molecular markers used in the present study helped to clarify the structure of the genetic diversity of the species. The wild germplasm collected from the forest areas in Ethiopia is already considered a valuable source of new diversity for breeding programs. For example, controlled crosses between wild Ethiopian progenitors and American cultivars (*i.e*. descendants from the Yemeni population) have produced high yielding F_1_ hybrids^44^, suggesting heterotic groups that can be further differentiated and optimized through targeted selection^45^. The genetic diversity of *C. arabica* available within the collections outside of Ethiopia is currently exploited for breeding programs to cope with the challenges of climate change around the world. While a global coffee genetic resource conservation consortium is certainly needed to ensure that existing coffee genetic resources are preserved in Ethiopia, the low level of genetic diversity in *C. arabica* compared to the much higher diversity in the present-day populations of its progenitors suggests that introgression events into the allotetraploid species from the diploid species such as those exploited to achieve coffee rust resistance in derivatives of the Timor hybrid^46,47^ are of paramount importance to broaden substantially the genetic diversity in the cultivated germplasm and increase environmental, economic and social sustainability of coffee cultivation.

## Conclusions

In our study we adopted a hierarchical sequencing approach to reduce the genome complexity and consequently to maximize the accuracy of sequence assembly of *Coffea arabica*, a tetraploid species, with the aim of reconstructing separately homoeologous sequences and provide a reference for studying allelic variation in Arabica. Our genome assembly is relatively fragmented, but it contains most of the conserved set of plant single-copy orthologs and allowed us to assign most of the predicted genes to their parental genome and to perform an in-depth unbiased analysis of Arabica population history and diversity. We found in *C. arabica* a very low level of genetic diversity and an extremely large fraction of private alleles, both when considering individual accessions as well as when comparing the polyploid species with the two progenitor species. All evidence collected are compatible with the origin of *C. arabica* from a single polyploidization event that occurred in very recent evolutionary times, as attested also by *in silico* forward simulations under different demographic scenarios.

Our results still reveal genetic differentiation between a group of ‘wild’ Ethiopian accessions and another group including other ‘wild’ Ethiopian accessions and most of cultivated accessions studied, including commercial germplasm belonging to the ‘Typica’ and/or ‘Bourbon’ lineages. The Ethiopian reservoir may be exploited for improvement of *C. arabica* cultivated varieties but, due to its limited divergence from the commercially grown materials, we suggest exploiting introgression events from the diploid parental species into the allotetraploid species, as in the case of the famous Timor hybrid. The extremely recent origin and low genetic diversity of *Coffea arabica* make many of the traditionally used approaches in plant breeding, trait mapping and gene isolation less efficient and the development of novel alternative approaches a definitive must.

## Methods

### Plant material for genome sequencing and annotation

Sequencing for genome assembly was performed using an individual of *C. arabica* ‘Bourbon Vermelho’. Seedlings were obtained by somatoclonal embryogenesis from cherries imported from a production area called Ahuachapan in El Salvador. Nine tissues/organs (young leaves, leaves, stems, roots, red drupes, green drupes, multiple drupes, meristems, buds) were sampled from the same variety. Sequencing for subgenome assignment was performed using an accession of *C. eugenioides* derived from an illycaffè greenhouse in Rivignano, Udine, Italy.

### Library construction for genome sequencing

A BAC library of 175,872 BAC clones was constructed from genomic DNA by Lucigen Corporation (Lucigen Corporation). 36,864 BACs were randomly selected, inoculated into 96 384-well plates and grown at 37 °C for 22 hours in 2x LB medium supplemented with chloramphenicol antibiotic. The 384 bacterial cultures of each plate were mixed in a single tube. Each pool was subjected to alkaline lysis of bacterial cells and BAC vector was amplified *in vitro* with Bacteriophage Phi29 polymerase (Illustra^TM^ TempliPhi^TM^ Large Construct V2 kit, Resnova) with an isothermic reaction at 20 °C for 16 hours. BAC DNA was then purified with ethanol and sodium-acetate precipitation and resuspended in distilled water. BAC pools were quantified on a fluorometer (Qubit, Invitrogen) and visualized on a 0.8% agarose gel in 1x TBE buffer. Illumina libraries were constructed with the Nextera^TM^ DNA Sample Preparation kit (New England Biolabs), following the manufacturer’s protocol.

Libraries were then purified with magnetic beads AMPure XP (Agencourt), quantified on a Caliper GX (Perkin Elmer), and sequenced using an Illumina HiSeq2000 (Illumina), generating 100-bp paired ends. Libraries from 12 pools were also sequenced with an Illumina MiSeq sequencer, generating 250-bp paired ends.

Whole-Genome Shotgun libraries were constructed from genomic DNA of the same individual of *C. arabica* and from one accession of *C. eugenioides* using the Illumina TruSeq DNA Sample prep kit, according to the manufacturer’s protocol. *C. arabica* WGS was performed using an Illumina HiSeq2000 and generated 100-bp paired ends. *C. eugenioides* WGS was performed using an Illumina HiSeq2000 and generated 125-bp paired ends.

For the same individual of *C. arabica* a 2-3 kbp mate-pair library was constructed using a Mate Pair Library v2 Sample Preparation kit following the Illumina protocol without gel electrophoresis size selection. The libraries were validated using a Bioanalyzer 2100 (Agilent), quantified using Qubit (Invitrogen) and then sequenced on Illumina HiSeq2000.

### Reads trimming and filtering

Reads were quality trimmed with erne-filter v1.4.3^48^ using default parameters and minimum read length of 50 bp. Adapters were removed with cutadapt^49^ using default parameters but -O 5 -n 2 -m 35. Cloning vector, *Escherichia coli* and chloroplast reads were filtered with erne-filter v1.4.3^48^. Mate pairs were trimmed and filtered using the same procedure as above and then sorted into genuine mate pairs or paired-ends with internally developed Perl scripts based on the presence/absence of the Biotine signature.

### *De novo* assembly, k-mer analysis and subgenome identification

Each BAC pool was assembled independently with ABySS v1.3.7^13^ with default parameters but k = 71, aligner=map, b = 1000000, p = 0.95, s = 500, n = 10. WGS mate pairs were used for scaffolding contigs within each BAC pool with SSPACE v3.0^50^ with a minimum of 10 mate pair links to join adjacent contigs. Repetitive DNA was masked with RepeatMasker with the following parameters: -qq -nolow -norna -no_is -gff with repeat library derived from the *C. canephora* genome^11^. k-mer analysis was carried out with Jellyfish^51^ with parameters -c 3 -s 10 G and -m either 16 or 51. Effective sequencing depth (N) was estimated with k-mer analysis based on modal k-mer frequency (M), read length (L), k-mer length (K) and on the formula N = M*L/(L-K + 1)^52^; genome size was derived from sequencing yield divided by N. For *C. canephora* k-mer analysis, reads were downloaded from NCBI Sequence Read Archive experiments ERX294808, ERX294809, ERX294819, ERX294831, ERX294847, ERX294857, ERX294862, ERX294873, ERX294881, and ERX294885. *C. eugenioides* and *C. canephora* 51-mers were generated from Illumina reads using Jellyfish^51^ with parameters -m 51 -c 3 -s 10 G. Scaffolds were classified with internally developed Perl scripts as belonging to either the canephora or eugenioides subgenome if they either shared more 51-mers with *C. canephora* or shared more 51-mers with *C. eugenioides*, respectively. Scaffolds with less than 1000 available 51-mers, *i.e*. either very short or containing mostly repetitive DNA, or with similar numbers of shared 51-mers for both parental species (difference < 10%) remained unclassified.

### RNA-Seq and transcript analysis

RNAs were extracted using Spectrum Plant Total RNA Kit (SIGMA) following the manufacturer’s protocol (www.sigmaaldrich.com/). 1.5 µg of good quality RNA (R.I.N. > 7) was used as starting material for library preparation with the Illumina mRNA-Seq Sample Prep kit v2.0 following the manufacturer’s instructions (www.illumina.com/). The poly-A mRNA was fragmented for 1.5 minutes at 94 °C and all purification steps were performed using 1X Agencourt AMPure XP beads. Library quality and quantity were assessed using the Agilent Bioanalyzer 2100 High Sensitivity and Qubit DNA High Sensitivity (Invitrogen) as described more in detail in^53^. Libraries were pooled together, and the obtained pool checked on an Agilent Bioanalyzer 2100 in order to determine the molarity. Paired-end sequencing was performed on the Illumina HiSeq2500 (www.illumina.com/systems/sequencing-platforms/hiseq-2500.html) generating 125-base reads.

Trimmomatic^54^ was used for adapter clipping and quality trimming. The minimum read length was set to 35 bp and a minimum quality score of 20 within a sliding window of 5. RNA-seq reads were aligned on the reference genome using hisat2^55^ using default parameters and setting the maximum intron length to 50 kbp. Genome-guided transcript reconstruction was performed independently for each RNA-library using stringtie^56^, setting the minimum junction coverage to 5 (option –j). The transcripts were further assembled using PASA^57^, a eukaryotic genome annotation tool that exploits spliced alignments of expressed transcript sequences to automatically model gene structures.

### Gene prediction

Gene prediction resulted integrating several sources of evidence: (i) RNA-seq data; (ii) nucleotide and protein alignments; (iii) *de novo* gene training and prediction. Five different programs were used for *ab initio* gene prediction: Augustus^58,59^, Snap^60^, Glimmer^61^ and GeneMark^62^. Intron coordinates derived from RNA-Seq read alignments were provided to GeneMark. Gene models generated by PASA were used to train Snap, Glimmer and Augustus. Briefly, the PASA alignment assemblies were used to automatically extract protein coding regions in order to generate a high-quality data set for training *ab initio* gene predictors. To discard the lowest quality gene models generated by PASA, from the training dataset, only complete genes and validated through a similarity search with blast against a dataset of *C. canephora* were considered. From the blast search, only proteins with a match with an e-value lower that 1e-^30^ and an alignment coverage higher than 90% were used to train the *ab initio* predictors.

Nucleotide and protein sequences ranging from close to distantly related organisms belonging to eudycotyledon taxonomic rank, Gentianales order, Coffea genus and *C. canephora* species were downloaded from NCBI and aligned to the reference genome using exonerate (https://www.ebi.ac.uk/about/vertebrate-genomics/software/exonerate). Only high-quality alignments were retained applying the following stringent criteria: 30% identity and 70% alignment coverage at the protein level, 50% identity and 70% alignment coverage at the nucleotide level.

Previously collected evidence was combined for gene prediction using EVidenceModeler^57^ in order to obtain a single gene model. EVidenceModeler (EVM) combines *ab initio* gene predictions and protein and transcript alignments into weighted consensus gene structures. To reduce false positive prediction and improve the overall gene prediction quality, several filters were applied:

1. Genes predicted only by *ab initio* programs were considered only if they were confirmed by at least two different *ab initio* programs, if they were complete (with a start and a stop codon) and longer than 300 base pairs.
2. Gene supported by external evidence (*e.g*. proteins/RNA-seq) were considered if confirmed by at least two different types of evidence or by one external evidence and one *ab initio* gene predictor.
3. Predicted genes with a low *ab initio* support (as per step 1) were further processed. Those supported by only one *ab initio* program were retained only if found in a database of Coffea protein sequences. While proteins with a sequence coverage match higher than 50% for both query and subject and an e-value lower than 1e-^6^ were recovered.

Gene models passing these filters were further processed using PASA to add UTR regions and predict alternative splicing.

To remove multiple isoforms of the same gene and sequence redundancy, DNA coding sequences were clustered using CD-HIT^63^ with option -g set to 1 and for each gene locus we selected the longest transcripts isoform. CD-HIT was initially run setting the clustering percentage identity to 0.9. A Perl script was developed to parse the output in order to empirically identify a threshold that clustered as many genes as possible while keeping subgenome k-mer classified genes in proper clusters. The optimal clustering percentage identity was obtained at 0.9961 (Fig. S5).

### Gene Annotation

BLASTp similarity searches (e-value threshold of 1e^−5^) of *C. arabica* predicted genes were performed against the non-redundant protein database (NCBI). InterProscan5^64^ was used to obtain the conserved protein domains and functional annotation. The databases used included PROSITE patterns, PRINTS, PFAM, PRODOM, SMART, TIGRFAM, and PANTHER. Gene Ontology and KEGG classifications were predicted running BLAST2GO 2.6.0^65^ on the BLASTp and InterProscan outputs.

### Plant material for genotyping

An extensive number of accessions from *C. arabica* (781), *C. canephora* (35) and *C. eugenioides* (10) were collected for genotypic analysis for this study. Because 45 of the *Coffea arabica* accessions failed to produce sufficient reads following GBS analysis they were removed from the study leaving a total of 736 Arabica accessions. The final *Coffea arabica* genotypes included in this study were represented as follows: 648 *C. arabica* accessions provided by CATIE and 88 *C. arabica* accessions collected in Yemen provided by Sana’a University. The *C. canephora* and *C. eugenioides* accessions were collected by IRD (Institut de Recherche pour le Développement, France)^66^ and provided by CATIE or CIRAD. The list of all 781 accessions is provided in Dataset S2.

In relation to this history, the Arabica accessions are coded using the following categories:

Survey Ethiopia: 441 accessions from the FAO survey in Ethiopia (FAO 1964), 84 from the ORSTOM survey (1966) in Ethiopia (Guillaumet 1967) and 16 accessions from the ‘Lejeune survey’ or other surveys collected in Ethiopia before 1957. In Dataset S2 geographic coordinates and altitude for 359 accessions provided by botanists during the FAO and ORSTOM surveys is provided.

Landrace cultivated: 49 accessions of Ethiopian cultivated populations that have been collected in Ethiopian farms in addition to the FAO and ORSTOM surveys. These surveys are less documented than the FAO and ORSTOM surveys.

Survey Yemen: 93 accessions representing subspontaneous-derived accessions cultivated in Yemen and collected by Sana’a University (88 accessions) or by FAO (5 accessions planted in the CATIE collection). Those accessions can be considered as domesticated. For 28 accessions provided by Sana’a University, the altitude and the geographical coordinates are reported in Dataset S2.

East Africa and Indian Old varieties: 45 accessions of varieties selected in the 30’s in India and East Africa.

Typica/Bourbon cultivars: Seven accessions from the CATIE field GeneBank that conformed to the botanical varieties described by Krug and Carvalho (1951). In this study they represent the two widely cultivated varieties in Asia and Latin America for more than two centuries.

### DNA extraction and genotyping

Leaves were collected and lyophilized, and genomic DNA was extracted using the ADNid method (http://www.adnid.fr/index-2-4A.html) at ADNid (Montpellier, France).

Genotyping by sequencing (GBS) was conducted at the Cornell University Institute for Genomic Diversity (http://www.igd.cornell.edu/index.cfm/page/GBS.htm). Illumina template libraries were produced using the restriction enzyme *Pst*I followed by single-end sequencing on a HiSeq2000 (Illumina) as previously described^67^.

### GBS analysis

787 *C. arabica* varieties along with 10 *C. eugenioides* and 35 *C. canephora* accessions were subjected to GBS using the restriction enzyme *Pst*I^67^. Ninety-six barcoded accessions were pooled, and each pool was run in one lane of a flow cell on an Illumina HiSeq2000 using a 91 bp single-end sequencing mode at the Institute for Genomic Diversity (IGD) at Cornell University. In total ~172 GB of DNA sequence data was obtained. Reads were initially processed to remove the individual barcodes corresponding to each accession and separated into individual fastq files for each accession using a custom python script. To avoid false positive SNP detection due to the allopolyploid nature of the *C. arabica* genome (*i.e*. polymorphism between the two subgenomes), two *in silico* reference sequences were generated, one for each of the two subgenomes. The canephora *in silico* reference was composed by (i) the assembled canephora subgenome, (ii) a full homoeologous complement of the assembled eugenioides subgenome, and (iii) the unassigned scaffolds. Similarly, the eugenioides *in silico* reference was composed by (i) the assembled eugenioides subgenome, (ii) a full homoeologous complement of the assembled canephora subgenome, and (iii) the unassigned scaffolds. In order to produce the full homoeologous complement of the assembled canephora subgenome, WGS reads of *C. arabica* ‘Bourbon Vermelho’ were aligned using BWA^68^ against the assembled canephora subgenome and the unassigned scaffolds. Then, homoeologous SNPs were called using GATK^69^ with default parameters and the alternative homoeologous reference was generated using GATK FastaAlternateReferenceMaker for sites with minimum depth 50 and allele frequency between 0.25 and 0.75. The same procedure was applied to obtain the full homoeologous complement of the assembled eugenioides subgenome.

GBS reads were aligned using BWA-MEM v0.7.10^68^ against the canephora and eugenioides *in silico* references in order to call allelic SNPs in the two subgenomes, respectively. For both artificial references, SNP calling was performed using Stacks v2.1^70^. Only variant sites covered by at least 10 reads were retained. Heterozygous SNPs with an allele frequency lower than 0.25 or higher than 0.75 were discarded. Polymorphisms detected using the two artificial references were merged and only variant sites called in native scaffolds of each subgenome were retained for subsequent analyses. To obtain a dataset of SNPs for STRUCTURE and PC analyses, Stacks was run with the option –r (minimum percentage of individuals in a population required to process a locus for that population) set to 0.75 and the option–max-clipped (maximum soft-clipping level, in fraction of read length) set to the default value of 0.20, corresponding to a minimum stack length of 73 bp. Variants sites with seven or eight missing genotype calls out of the eight Bourbon/Typica accessions were interpreted as misalignments and filtered out.

Principal Component Analysis was performed using the R package ade4^71^. A hierarchical study of the diversity has been conducted using a model-based clustering procedure with admixture as implemented in STRUCTURE v2.3.4^72^. Runs were performed at values of *k* ranging from 1 to 13 with 10 replicates per K and a burn-in period of 75,000 and 75,000 MCMC repetitions. Plotting *k* vs ΔK indicated that the highest value was for 2 groups followed by 3 groups (Fig. S2). Hence, after applying a threshold of 0.80 of membership, STRUCTURE was performed again on each of the 2 populations. According to ΔK, the second population was divided in 2 groups (Fig. S2). A Principal Coordinates Analysis (PCoA) was performed in R (version 3.5.1, 64 bit; www.r-project.org/) with ape^73^, using provesti distance matrix calculated using poppr package^74^. The PCoA was plotted with ggplot2 (Fig. S3).

Given the low level of diversity of the species and the high frequency of the *PstI* restriction sites, we increased genome sampling for a better estimation of π, Tajima’s *D* and private or shared SNPs in *C. arabica*, *C. canephora*, and *C. eugenioides* by running Stacks with the option –r (minimum percentage of individuals in a population required to process a locus for that population) set to 0.50 and the option–max-clipped (maximum soft-clipping level, in fraction of read length) set to the value of 0.68, corresponding to a minimum stack length of 30 bp. For these analyses, sites in which all *C. eugenioides* accessions were homozygous reference and all *C. canephora* accessions were homozygous alternative, or vice versa, were interpreted as residual homoeologous SNPs and filtered out, as well as sites within missing data in *C. eugenioides* and *C. canephora* and >95% heterozygous calls in *C. arabica*. Nucleotide diversity and Tajima’s *D* were calculated for each group using the diversity.stats method included in the R package PopGenome^75^.

## Supplementary information

Supplementary information.

## Data Availability

The data have been deposited with links to BioProject accession number PRJNA554647 in the NCBI BioProject database (https://www.ncbi.nlm.nih.gov/bioproject/). The genome sequence is available at https://worldcoffeeresearch.org/work/coffea-arabica-genome/.
