# Supplementary material for: A single polyploidization event at the origin of the tetraploid genome of Coffea arabica is responsible for the extremely low genetic variation in wild and cultivated germplasm
Source: Sci Rep. 2020 Mar 13;10:4642. doi: 10.1038/s41598-020-61216-7 (PMC7069947; doi:10.1038/s41598-020-61216-7)
Supplement: Supplementary file 1 — Supplementary information. [file 41598_2020_61216_MOESM1_ESM.pdf]

Supplemental text

Gene prediction

The raw gene prediction produced a total of 101,644 genes. As described in material and methods, we applied several filters to reduce the number of false positive predictions. 67.9% of the genes passed the quality filters, while 1.9% of the genes were discarded because too short (length < 300 bp), 1.9% because of a low evidence support and 28.7% because with a low ab-initio support. Genes with a low ab-initio support were further processed and searched for similarity against C. canephora proteome allowing the rescue of further 9,351 genes. After the last step with PASA, for UTRs and alternative splicing prediction, the final dataset had a total of 78,311 protein-coding genes and 93,078 transcripts. Similarity search against NR database shows that 97.1% of the transcripts present at least a significant match, suggesting that the filters applied to the gene predictions allowed to eliminate the majority of wrong predictions. Using Blast2GO, we were able to assign 5,841 distinct gene ontology terms to 82% of the transcripts: 48.3% of the terms belonged to biological process ontology, 39.2% to molecular function and 12.5% to cellular component. Predicted genes cover 92.4% of the plant orthologs set of BUSCO, suggesting a high completeness of the prediction.

To remove the redundancy due to assembly strategy, we performed a sequence clustering using CD-HIT. We obtained a final number of 46,562 clusters, 21,254 containing as cluster representative sequence a gene assigned to the canephora parental genome, 22,888 with a representative gene assigned to the eugenioides parental genome, while 2,420 with a gene that was not assigned to any parental genome. We found that 91.4% of the clusters are composed by genes from the same parental genomes, while only 8.6% of the clusters are composed by genes from the two genomes, indicating that the identity threshold used was able to discriminate between genes from the two parental genomes. Finally, 62.5% of

the clusters are singleton, *i.e.* they are composed by just one sequence, while 20% are composed by two sequences. The number of clusters containing more than 5 sequences is only 1.3%.

Genetic diversity structuring in *Coffea Arabica*

After running STRUCTURE on the whole dataset as presented in the paper, we run STRUCTURE only on G1 and assigned the color codes on the PCA according to the new ancestry assignments. This further analysis revealed a clear structuration of the Ethiopian germplasm (Figure S2, Figure S3A, Figure S3B). The Fixation Index revealed genetic differentiation between two Ethiopian populations (F_{ST} = 0.34), which were coded G1A and G1B. Each Ethiopian population showed genetic differentiation with the Yemeni population (F_{ST} = 0.54, G1A vs. G2; F_{ST} = 0.65, G1B vs. G2). We mapped the geographical sites of the FAO and ORSTOM surveys and compared the location of sampling with genetic variation explained by the population structure (Fig. 2F, Figure S3B, Table S3). We propose to rename the G2 population as the ‘Harar-Yemen’ group as it included Ethiopian germplasm from the eastern region around the city of Harar and all the Yemeni varieties (Fig. 2F). The population G1B included all accessions from the Sheka forest, 91% of the accessions collected from Mizan-Teferi and 92% of those collected from Teppi (Table S3), around a rainforest area that is located approximately 200 km west of the city of Jimma. We hence propose to call this population “Sheka”.

We found that all accessions collected around Jimma at the sites of Agaro, Bonga, the Didessa wildlife sanctuary, Shebe, Gera and Wush-Wush were assigned to the G1A population (Table S3). Some accessions that were naturally found further east across the Rift valley in the region of Sidamo (Yirga Cheffe) were also grouped into G1A.

Therefore, population G1A includes germplasm dispersed over a vast region around Jimma delimited northwards by the Diddessa wildlife sanctuary and southwards by Maji, Shebe, Bonga, Wush-Wush, and the Bonga forest. In this mountainous area, the natural forest has been interspersed with patches of small coffee plantations since the time the surveys were made. The diversity of habitats included forest edges, agricultural fields, graze land and swamps. We propose to call this population ‘Jimma-Bonga’.

We also classified individuals within the three populations according to the plant material category assigned by FAO and ORSTOM reports (Figure S4). The categories ‘Forest’, ‘Garden’, and ‘Intensive plantation’ were adopted by the FAO (1964-1965) and ORSTOM (1966) surveys to refer to material collected in the wilderness, around human settlements, or in large scale plantations. In this study we considered that ‘wild’ accessions originated from forest and garden at the time of the survey except if the provenance of the seeds was clearly cited, as for example was the case of some accessions for which the authors mentioned ‘Harar’ or ‘Kenya’. At the time of the surveys, 92% of the ‘Harar-Yemen’ population (G2) was classified as ‘intensive plantation’ by the botanists, whereas only 15% of the Jimma-Bonga population (G1A) and no accessions of the Sheka population (G1B) were categorized as such. As much as 63% of the Sheka population and 27% for the Jimma-Bonga population were previously classified as forest accessions (Figure S4).

The species structure in the three main populations was well supported by geographic background information. For the first time we can propose a genetic diversity structuring of the species based on three populations that include mainly Yemeni, East Africa and Indian cultivated genotypes in the population ‘Harar-Yemen’ (G2), Ethiopian ‘wild’ and

landraces in the population 'Jimma-Bonga' (G1A) and mainly Ethiopian 'wild' genotypes in the population 'Sheka' (G1B). The 'Harar-Yemen' population located at the east of the African continent was geographically closer to the 'Jimma-Bonga' population (G1A) than the 'Sheka' population (G1B), which is located more southwestern in Ethiopia and consists of the most feral accessions. Previous analysis of phenotypic traits from 148 accessions of *C. arabica* differentiated them into two main groups, Ethiopian accessions collected west of the Great Rift Valley and commonly cultivated varieties and Ethiopian accessions collected east of the Great Rift Valley (Montagnon and Bouharmont 1996).

In the present study we observed little differentiation between Rift Valley southwest and southeast populations and the genetic data does not support differentiation of the collection between the two sides of the Great Rift Valley as observed in previous studies. Our study, however, mainly takes into account accessions west of the Rift Valley. It would be interesting to include more accessions east of the Great Rift Valley, most notably from the Harenna forest (Bale) to determine whether a fourth population based on Harenna forest accessions could be identified as suggested by the preliminary work of Aga et al. (2003). As stated by Davis et al. (2012), the largest and most diverse populations of indigenous (wild) *C. arabica* occur in the highlands of southwestern Ethiopia, but the native range includes satellite populations in southeastern South Sudan (Boma Plateau) and northern Kenya (Mt Marsabit). It would be quite informative to include those satellite populations in future studies.

Aga E, Bryngelsson T, Bekele E, Salomon B. 2003. Genetic diversity of forest arabica coffee (Coffea arabica L.) in Ethiopia as revealed by random amplified polymorphic DNA (RAPD). *Hereditas* **138**: 36–46.

Davis AP, Gole TW, Baena S, Moat J. 2012. The Impact of Climate Change on Indigenous Arabica Coffee (Coffea arabica): Predicting Future Trends and Identifying Priorities. *PLoS One* **7**: 1–13.
Montagnon C, Bouharmont P. 1996. Multivariate analysis of phenotypic diversity of *Coffea arabica*. *Genetic Resources and Crop Evolution* **43:** 221–227.

Fig. S1. 16-mer analysis of paired-end WGS sequences. On the x axis the k-mers coverage, on the y axis the abundance of k-mers. When the two subgenomes are different they provide the expected coverage at ~54X, while identical 16-mers from homoeologous regions provide a double coverage at ~108X.

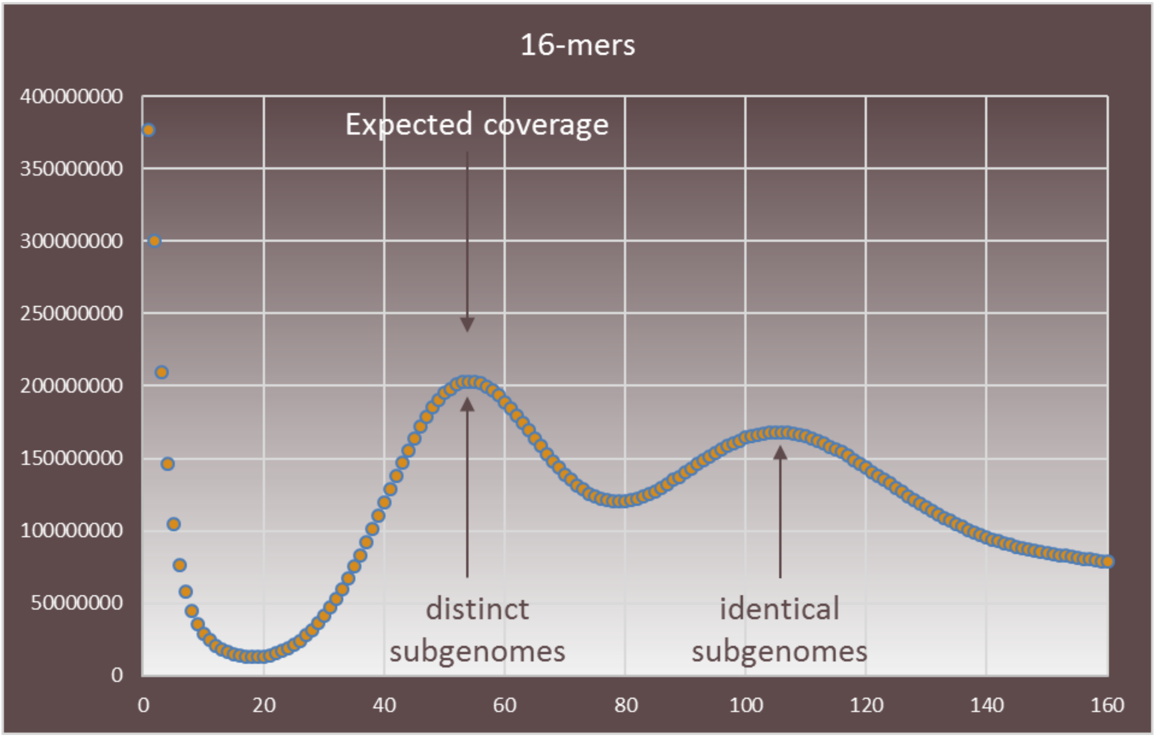

Fig. S2. STRUCTURE analysis. Evanno graph for A. Full Arabica dataset. B. Population 1 (G1). C. Population 2 (G2)

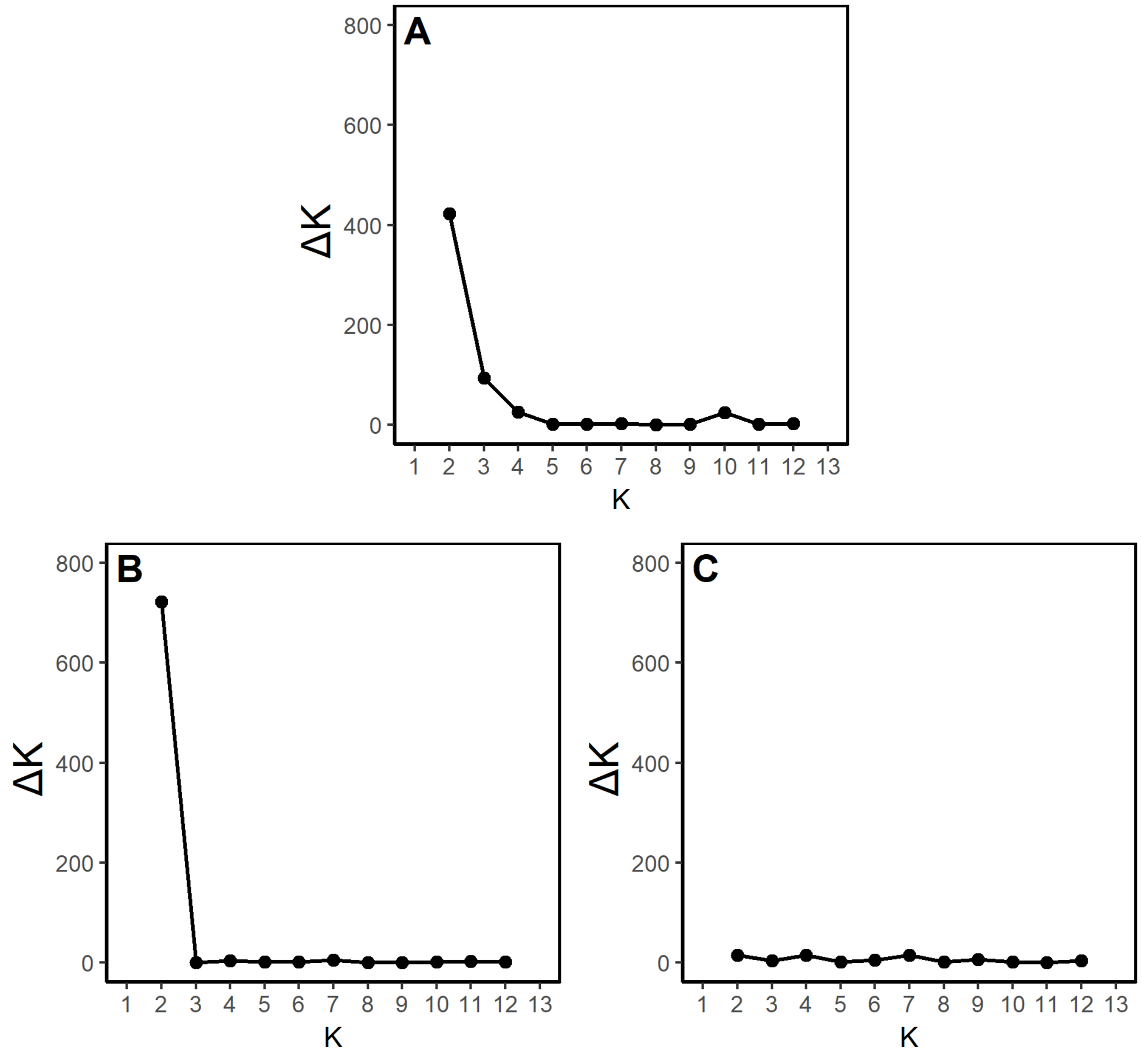

Figure S3. Principal Coordinate Analysis of the G1 group of the Arabica population in relation to ancestry assignment provided by STRUCTURE. Accessions from G1 subgroup of fig. 2E in the main text are plotted in panel A. Map of Ethiopia (panel B) showing the locations of the coffee accessions as a function of their STRUCTURE population (see supplemental section: Genetic diversity structuring in *Coffea arabica*)

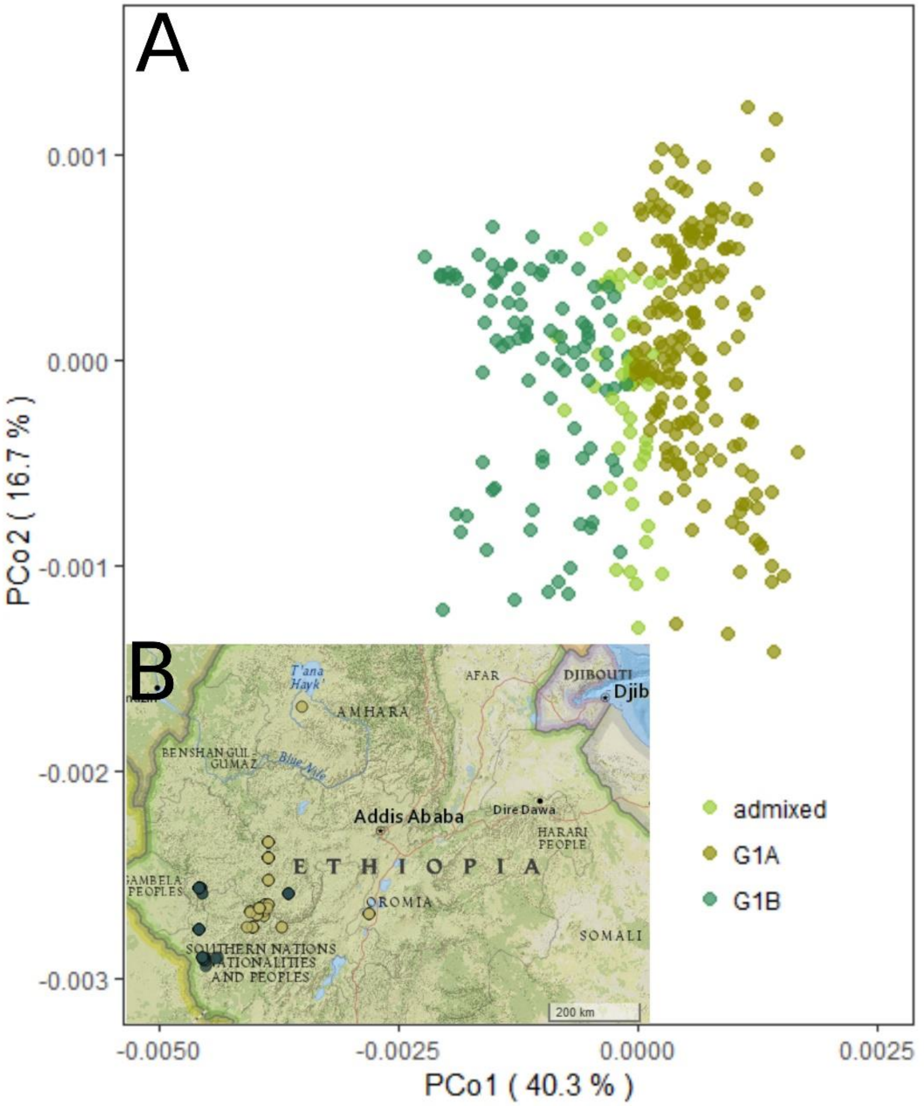

Figure S4. Distribution of accessions of the three subpopulations according to their status at the time of the FAO and ORSTOM surveys. The categories 'Intensive plantation', 'Garden' and 'Forest' correspond to categories adopted by the FAO (1964-1965) and the ORSTOM (1966) surveys. Intensive plantations refer to coffee cultivated after land clearing with systematic soil preparation and seedling planting and managed in order to maximize the volume of production and productivity. Garden represents coffee grown in smallholdings under a few shade trees usually combined with other crops and fruit trees. Forest represents coffee grown in forests (or semi-forested) areas where eventually farmers slash weeds, lianas and competing shrubs, thin forest trees and fill open spaces with local seedlings (Labouisse and Kotecha 2008). In this study we considered that the 'wild' accessions came from forest and garden at the time of the survey except if the provenance of the seeds was mentioned as was the case for example for some accessions for which the authors mentioned 'Harar' or 'Kenya'.

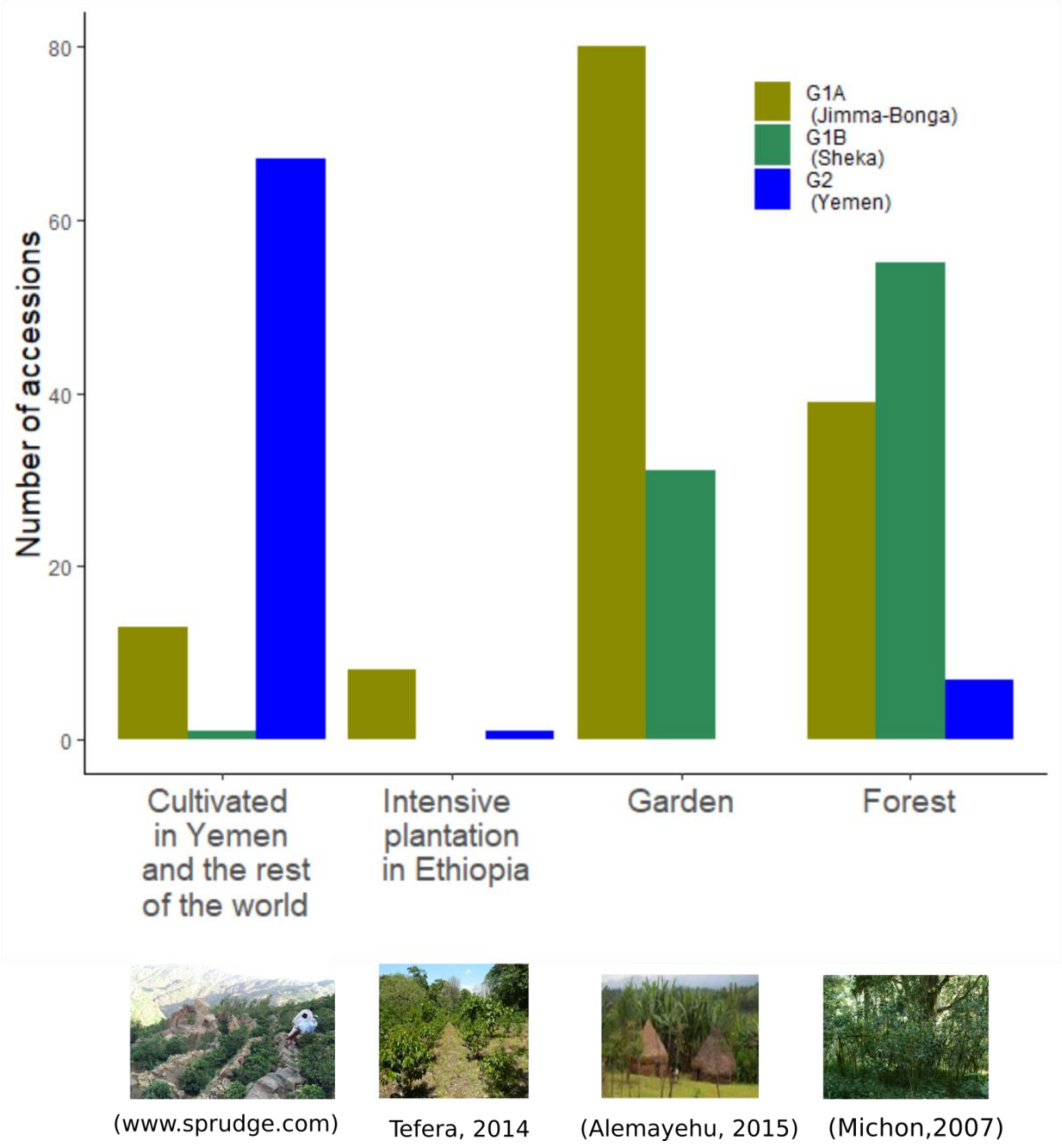

Figure S5. Identification of the optimal threshold of sequence identity to collapse duplicates using the CD-HIT software. DNA coding sequences were clustered using CD-HIT. CD-HIT was initially run with the default clustering percentage identity of 0.9. Genes are classified by CD-HIT as representative or non-representative within each cluster. In order to identify a threshold for collapsing redundant gene sequences of the same homoeolog while separating inter-homoeologous gene sequences, we plotted for each bin of sequence identity (%) the number of sequences in each cluster consistent with the subgenome assignment of the representative gene of that cluster (in blue) versus the number of sequences in each cluster inconsistent with the subgenome assignment of the representative gene of that cluster (in orange). We then identified empirically the percentage of identity (0.9961) representing a tradeoff between sensitivity and specificity.

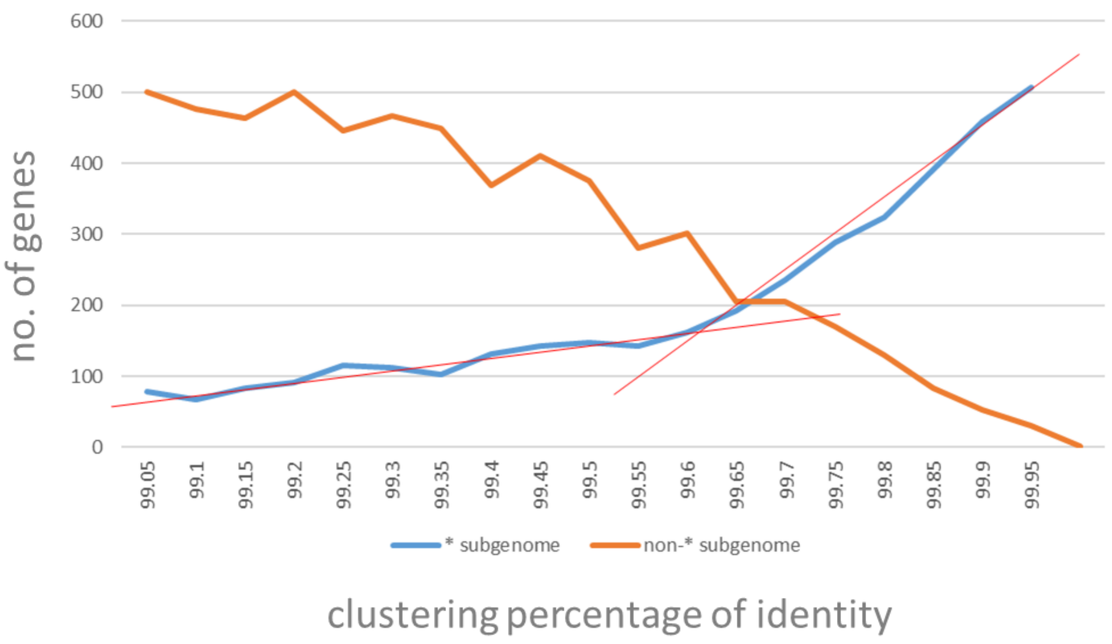

Table S1. Sequencing of *C. arabica*, *C. eugenioides,* and *C. canephora*: Whole Genome, BAC pools, RNA-seq, dRAD-seq. For WGS sequencing, total nucleotides and coverage are reported. For pool sequencing, total number of sequenced BACs, number of sequenced pools, number of BACs per pool, average BAC insert size, physical coverage of BACs sequenced, and total sequencing are reported. For RNA-seq of *C. arabica*, different tissues/organs sequenced (replicates are reported in multiple raws), millions of reads and total sequencing are reported. For dRAD-seq, number of accessions, millions of reads and total sequencing are reported.

**WGS *C. arabica***	**Insert size (bp)**	**Read length (bp)**	**Nucleotides (Gb)**	**Coverage**
Miseq overlapped	420	300+300	22.5	17.3x
Hiseq overlapped	180	100+100	34.4	26.5x
Hiseq	250	100+100	8.2	6.3x
Hiseq long	900	100+100	20.2	15.5x
Mate pairs	2000	100+100	42.7	32.8x
**TOTAL**			**128.0**	**98.4x**

**WGS *C. eugenioides***	**Insert size (bp)**	**Read length (bp)**	**Nucleotides (Gb)**	**Coverage**
Hiseq 250bp	370	250+250	29.1	41.6x
Hiseq 125bp	370	125+125	9.1	13.0x
**TOTAL**			**38.2**	**54.6x**

**BAC *C. arabica***	**Pools**	**BAC x pool**	**Insert size (kb)**	**Physical Coverage**	**Nucleotides (Gb)**
36,864	96	384	≈100	2.84x	488

***C. arabica* tissue**	**Insert size (bp)**	**Read length (bp)**	**Reads (millions)**	**Nucleotides (Gb)**
Stem	213	125+125	40.38	5.05
Stem	215	125+125	55.62	6.95
Green drupe	228	125+125	54.41	6.80
Red drupe	222	125+125	45.23	5.65
Red drupe	222	125+125	49.51	6.19
Multiple drupes	222	125+125	41.74	5.22
Root	222	125+125	39.58	4.95
Root	222	125+125	52.04	6.51
Young leaf	222	125+125	50.70	6.34
Young leaf	218	125+125	48.51	6.06

Bud	222	125+125	56.49	7.06
Meristem	222	125+125	25.85	3.23
**TOTAL**			**560.06**	**70.01**

**dRAD**	**Accessions**	**Read length (bp)**	**Reads (millions)**	**Nucleotides (Gb)**
C. arabica	736	91	1,811.3	164.83
C. canephora	35	91	89.3	8.13
C. eugenioides	10	91	27.3	2.48
**TOTAL**			**1,927.9**	**175.44**

Table S2. Separation of the Arabica accessions (Survey Ethiopia, Survey Yemen, Bourbon/Typica, Landrace cultivated, Indian old varieties, East Africa old varieties) as calculated by the software STRUCTURE.

	G1A	G1B	G2	Number of accession by category
**Yemeni descending varieties**				
Bourbon/Typica	0	0	7	7
Survey Yemen	0	0	92	92
East African old varieties	0	1	33	34
Indian old varieties	0	0	5	5
**Ethiopian material**				
Landrace cultivated	13	0	22	35
Survey Ethiopia	170	92	27	289
**Total**	**183**	**93**	**186**	**462**

Table S3. Survey sites of Ethiopian accessions (surveys FAO, ORSTOM and Lejeune). G1A is mainly located near Bonga, Didessa Wildlife sanctuary, Jimma, Gera, Shebe and Wush-Wush,while G1B is mainly located near Mizan-Teferi, Teppi and Sheka forest (today part of the Bench Maji Zone).

**Collection sites**	**G1A**	**G1B**
Agaro	29	
Bada Buna	22	12
Bonga	27	
Decchia	1	
Didessa Wildlife sanctuary	31	
Geisha mount	1	1
Gera	13	
Ghembi	2	
Gojeb	1	
Gore	1	
Goré	1	
Harar	1	
Jimma	9	
Kossa	1	
Limu	2	
Maji		1
Mizan Teferi	2	20
Shebe	20	
Sheka forest		12
Suntu	1	
Tippi	4	44
Tippi-Goré		2
wush-wush	8	
Zeghie	3	
**Total**	**180**	**92**

Table S4. Values of nucleotide diversity for crop species in literature reports.

Crop species	Π	Reference
Apple	2.2×10^{-3}	Duan et al. 2017
Bread wheat	$1.5 \times 10^{-4} – 5.7 \times 10^{-4}$	Akhunov et al. 2010
Cassava	2.6×10^{-3}	Kawuki et al. 2009
Cucumber	3.2×10^{-3}	Qi et al. 2013
Date palm	9.2×10^{-3}	Hazzouri et al. 2015
Grapevine	5.5×10^{-3}	Liang et al. 2019
Peach	1.5×10^{-3}	Cao et al. 2014
Potato	1.1×10^{-2}	Hardigan et al. 2017
Rice	$6 \times 10^{-4} – 1.6 \times 10^{-3}$	Huang et al. 2012
Soybean	1.89×10^{-3}	Lam et al. 2010
Tomato	$2.49\times10^{-4} – 2.81\times10^{-3}$	Causse et al. 2013
Watermelon	1.4×10^{-3}	Guo et al. 2013

References of Table S4

Akhunov, E.D., Akhunova, A.R., Anderson, O.D., et al. (2010) Nucleotide diversity maps reveal variation in diversity among wheat genomes and chromosomes. *BMC Genomics*, **11**, 702.

Cao, K., Zheng, Z., Wang, L., et al. (2014) Comparative population genomics reveals the domestication history of the peach, Prunus persica, and human influences on perennial fruit crops. *Genome Biol.*, **15**, 415.

Causse, M., Desplat, N., Pascual, L., et al. (2013) Whole genome resequencing in tomato reveals variation associated with introgression and breeding events. *BMC Genomics*, **14**, 791.

Duan, N., Bai, Y., Sun, H., et al. (2017) Genome re-sequencing reveals the history of apple and supports a two-stage model for fruit enlargement. *Nat. Commun.*, **8**, 249.

Guo, S., Zhang, J., Sun, H., et al. (2013) The draft genome of watermelon (Citrullus lanatus) and resequencing of 20 diverse accessions. *Nat. Genet.*, **45**, 51–58.

Hardigan, M.A., Laimbeer, F.P.E., Newton, L., et al. (2017) Genome diversity of tuber-bearing Solanum uncovers complex evolutionary history and targets of domestication in the cultivated potato. *Proc. Natl. Acad. Sci. U. S. A.*, **114**, E9999–E10008.

Hazzouri, K.M., Flowers, J.M., Visser, H.J., et al. (2015) Whole genome re-sequencing of date palms yields insights into diversification of a fruit tree crop. *Nat. Commun.*, **6**, 8824.

Huang, X., Zhao, Y., Wei, X., et al. (2012) Genome-wide association study of flowering time and grain yield traits in a worldwide collection of rice germplasm. *Nat. Genet.*, **44**, 32–39.

Kawuki, R.S., Ferguson, M., Labuschagne, M., Herselman, L. and Kim, D.-J. (2009) Identification, characterisation and application of single nucleotide polymorphisms

for diversity assessment in cassava (Manihot esculenta Crantz). *Mol. Breed.*, **23**, 669–684.
Lam, H.-M., Xu, X., Liu, X., et al. (2010) Resequencing of 31 wild and cultivated soybean genomes identifies patterns of genetic diversity and selection. *Nat. Genet.*, **42**, 1053–1059.
Liang, Z., Duan, S., Sheng, J., et al. (2019) Whole-genome resequencing of 472 Vitis accessions for grapevine diversity and demographic history analyses. *Nat. Commun.*, **10**, 1190.
Qi, J., Liu, X., Shen, D., et al. (2013) A genomic variation map provides insights into the genetic basis of cucumber domestication and diversity. *Nat. Genet.*, **45**, 1510–1515.

Dataset S1 (separate file). Predicted levels of nucleotide diversity (π) and percentage of private SNPs under the hypothesis of a single hybridization event occurring 10,000 years before present and population growth to the current size (Ne=10,000) within 1,000 years after hybridization or at constant rate until present

Dataset S2 (separate file). List of accessions subjected to genotyping by sequencing with geographic coordinates and altitude from the FAO and ORSTOM surveys if available. Geographic coordinates of 30 Yemen accessions are also provided.
